# Development of an Effective 6-Methylpurine Counterselection Marker for Genetic Manipulation in *Thermococcus barophilus*

**DOI:** 10.3390/genes9020077

**Published:** 2018-02-07

**Authors:** Tiphaine Birien, Axel Thiel, Ghislaine Henneke, Didier Flament, Yann Moalic, Mohamed Jebbar

**Affiliations:** 1 Université de Brest (UBO, UBL), Institut Universitaire Européen de la Mer (IUEM)–UMR 6197, Laboratoire de Microbiologie des Environnements Extrêmes (LM2E), Rue Dumont d’Urville, F-29280 Plouzané, France; Tiphaine.Birien@univ-brest.fr (T.B.); taxelt@gmail.com (A.T.); Ghislaine.Henneke@ifremer.fr (G.H.); Didier.Flament@ifremer.fr (D.F.); ymoalic@univ-brest.fr (Y.M.); 2 CNRS, IUEM–UMR 6197, Laboratoire de Microbiologie des Environnements Extrêmes (LM2E), Rue Dumont d’Urville, F-29280 Plouzané, France; 3 Ifremer, UMR 6197, Laboratoire de Microbiologie des Environnements Extrêmes (LM2E), Technopôle Pointe du diable, F-29280 Plouzané, France

**Keywords:** archaea, piezophiles, hyperthermophiles, genetics, gene deletion, deep sea, hydrothermal vents

## Abstract

A gene disruption system for *Thermococcus barophilus* was developed using simvastatin (HMG-CoA reductase encoding gene) for positive selection and 5-Fluoroorotic acid (5-FOA), a *pyrF* gene for negative selection. Multiple gene mutants were constructed with this system, which offers the possibility of complementation in trans, but produces many false positives (<80%). To significantly reduce the rate of false positives, we used another counterselective marker, 6-methylpurine (6-MP), a toxic analog of adenine developed in *Thermococcus kodakarensis*, consistently correlated with the *TK0664* gene (encoding a hypoxanthine-guanine phosphoribosyl-transferase). We thus replaced *pyrF* by *TK0664* on our suicide vector and tested *T. barophilus* strain sensitivity to 6-MP before and after transformation. Wild-Type (WT) *T. barophilus* is less sensitive to 6-MP than WT *T. kodakarensis*, and an increase of cell resistance was achieved after deletion of the *T. barophilus* *TERMP_00517* gene homologous to *T. kodakarensis* *TK0664*. Results confirmed the natural resistance of *T. barophilus* to 6-MP and show that *TK0664* can confer sensitivity. This new counterselection system vastly improves genetic manipulations in *T. barophilus* MP, with a strong decrease in false positives to <15%. Using this genetic tool, we have started to investigate the functions of several genes involved in genomic maintenance (e.g., *pol*B and *rnh*B).

## 1. Introduction

The Thermococcales are generally found in natural biotopes typical of thermophilic microorganisms. Originally discovered in terrestrial and submarine hot vents, they have since been found in deep subsurface environments [1,2]. The Thermococcales order is presently represented by three genera: *Pyrococcus* [3], *Thermococcus* [4] and *Palaeococcus* [5,6]. Thermococcales are getting increasing attention from academia and industry because they provide a unique source of stable biocatalysts and other products such as archaeal lipids and compatible solutes [7,8]. Several species are used as biological models or sources of thermostable molecules studied in structural and metabolic biochemistry, genetics, and microbial physiology [9].

Members of this order grow easily under anaerobic conditions in the laboratory, making them models of choice for many years in fundamental or applied research projects [10,11]. Notably, their metabolism is based on the degradation of peptide and carbohydrate substrates to produce organic acids, hydrogen gas (H_2_) and carbon dioxide (CO_2_). This is associated with the production of hydrogen sulfide through the reduction of elemental sulfur [12]. The production of H_2_ has been well characterized for different models of Thermococcales even when elemental sulfur (S°) is not supplied in the growth medium [13,14,15,16].

*Thermococcus barophilus* is a hyperthermophilic, piezophilic and heterotrophic archaeon belonging to the Thermococcales order and Euryarchaeota phylum. *T. barophilus* was isolated from a deep-sea hydrothermal vent in 1993 and has the particularity of being the first true piezophile characterized. Indeed, it grows optimally at 85 °C with a pressure of 40 MPa. However, *T. barophilus* can also grow in a range spanning from atmospheric pressure (0.1 MPa) up to 85 MPa [17]. These features make it a good model for studying piezophilic and stress responses, and adaptive mechanisms [18,19,20].

Although comparative genomic and transcriptomic experiments offer substantial information about overall adaptive responses to changes in physico-chemical environmental conditions, the fundamental biological processes of cellular systems require synergy between biochemical and genetic approaches. Thus, as for eukarya and bacteria, genetic techniques have been developed for archaea, but have been seriously held back by limitations of culture conditions, expertise and the availability of suitable genetic markers for screening methods [21,22,23,24,25].

Nevertheless, genetic experimentation is now expanding on four groups of Archaea: Methanogens, Halophiles, Thermococcales and Sulfolobales, with more genetic manipulations being performed in Euryarchaeota than Crenarcheota [23].

Since the first genetic system developed in *Thermococcus kodakarensis* [26], several studies have been published showing that genetic manipulation is possible in different species of Thermococcales (e.g., *Pyrococcus furiosus*, *Pyrococcus yayanosii*, *Thermococcus kodakarensis* and *Thermococcus onnurineus*) [27,28,29,30]. Among these, a gene deletion system has recently been developed for the *T. barophilus* MP Δ*pyrF* host, relying on simvastatin resistance and 5-fluoroorotic acid sensitivity [31]. In this system, a suicide vector carries the selection markers, HMG-CoA reductase and *PyrF* genes, and the flanking areas of a targeted gene that are used in a double-crossover recombination to: (i) integrate in the chromosome; and then (ii) disrupt the gene of interest.

Indeed, two configurations are possible, either obtaining the mutant deleted in the targeted gene or a return to the wild-type state. However, as described previously when using the *PyrF*/5-FOA marker for counterselection [32], a third configuration is also obtained with a high rate of false positives (up to 80%), probably due to a lack of efficiency of the antibiotic, since no mutation was detected in *PyrF* alleles [31].

To avoid the stage of false-positive screening, which is particularly time-consuming for our model (at least five days of incubation), we improved our genetic tool by using another counterselective marker, 6-methylpurine (6-MP). This toxic compound is an adenine analog that depresses the growth of different organisms [33,34,35]. It inhibits the formation of guanylic acid from adenylic acid, decreases the incorporation of adenine into ribonucleic acid, and inhibits ribosome synthesis [36].

In *T. kodakarensis*, 6-MP is known as an effective growth inhibitor consistently correlated with *TK0664*, a gene encoding a hypoxanthine-guanine phosphoribosyl-transferase [37]. Thus, in this study, we replaced *pyrF* by *TK0664* on our suicide vector and tested the inhibitory effect of 6-MP on growth of several strains of *T. barophilus* before and after transformation with a suicide vector bearing sensitivity to 6-MP.

This new counterselection system strongly improves genetic manipulations in *T. barophilus* MP, with a strong decrease in the rate of false positives to 15%. Among the deletion mutants obtained, we confirmed that *polB* or *rnhB* could be deleted from *T. barophilus* without any effect of these individual deletions on cell growth under our growth conditions. This is similar to observations in *T. kodakarensis* [38,39] and showed that PolD is sufficient for DNA replication in *T. barophilus*, as shown in *T. kodakarensis* [39].

## 2. Materials and Methods

### 2.1. Strains and Growth Media

The strains used in this study are described in Table 1. All strains were grown under anaerobic conditions, at 85 °C in Thermococcales Rich Medium (TRM). [40], which was made up as follows: 1 L of demineralized water was supplemented with 23 g NaCl, 5 g MgCl_2_, 3.3 g PIPES, 4 g Tryptone, 1 g yeast extract, 0.7 g KCl, 0.5 g (NH_4_)_2_SO_4_, 0.05 g NaBr, 0.01 g SrCl_2_, and 1 mL resazurin 1%. The pH was adjusted to 6.6–6.7 and the medium autoclaved for 20 min at 121 °C. Once cooled, 1 mL of each of the following solutions was added sterilely: 5% k_2_HPO_4_, 5% KH_2_PO_4_, 2% CaCl_2_ 2H_2_O, 10 mM Na_2_W0_4_, 25 mM FeCl_3_ 6H_2_0. The liquid medium was dispensed in 50 or 100 mL vials, sulfur was added (2 g/L), and all vials were sealed with butyl-rubber stoppers, vacuum and N_2_ gas addition steps were required to remove O_2_ and to maintain the culture media under anaerobic conditions. The liquid phase was reduced by 0.1 mL of a Na_2_S, 9H_2_0 solution. TRM was used in liquid or solid form and, for the latter case, 10 g/L of phytagel (Sigma-Aldrich Chimie, L’Isle D’Abeau Chesnes, St Quentin Falavier, France) were added to the TRM liquid medium.

After cell transformation, the transformants were selected on TRM supplemented with 2.5 µg/mL of simvastatin (Sigma) or 100 µM of 6-MP (Sigma).

### 2.2. Microbial Growth

Pre-cultures were incubated overnight at 85 °C for 16 h, an aliquot of 0.2 mL of each overnight culture was introduced into 20 mL of fresh TRM supplemented with different concentrations of 6-MP (0 µM, 10 µM, 50 µM, 100 µM and 250 µM). Cultures were incubated at 85 °C and cell numbers were counted every 3 h. Growth was monitored by cell counting using a Thoma chamber and photonic microscopy at a magnification of 40× (Olympus) or using flow cytometry (CyFlowSpace, Sysmex Partec, GmbH, Görlitz, Germany). Cells were fixed with 2.5% glutaraldehyde (Sigma) and counted by one of the two cell counting methods described above. All the experiments were carried out in triplicate.

### 2.3. Construction of Gene Deletion Vectors

**Figure 1:**
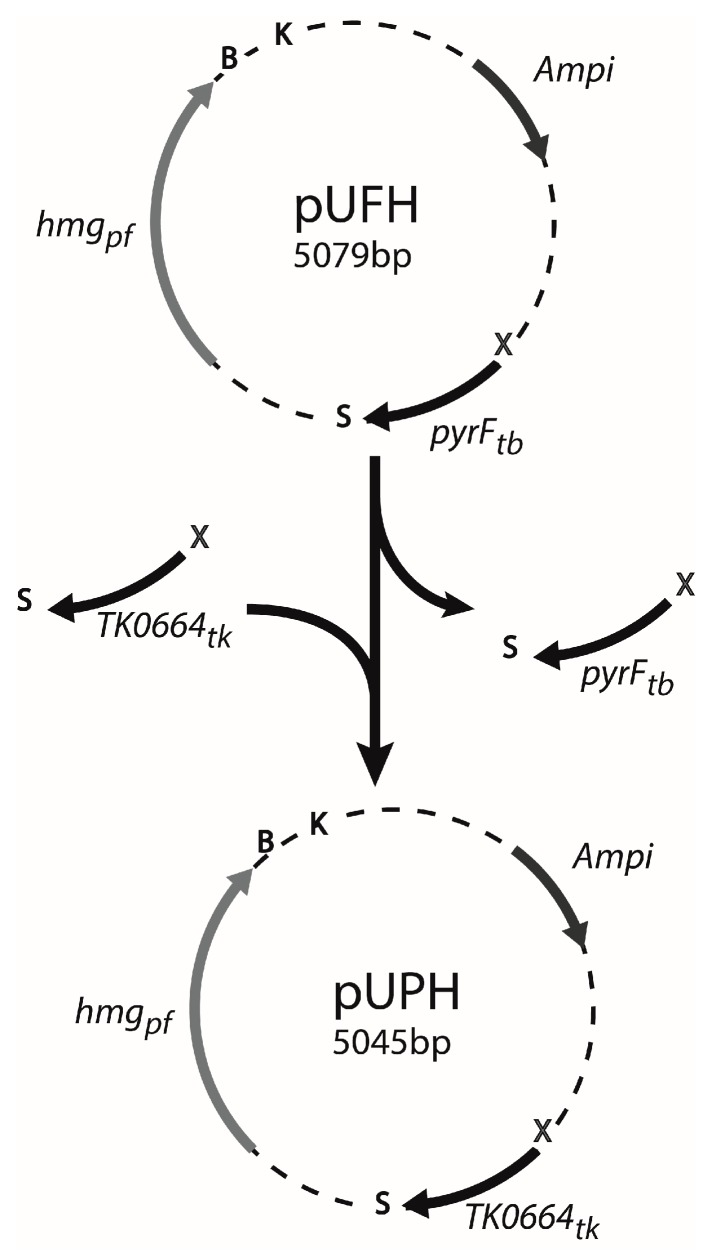
Construction of the pUPH plasmid. Primers XhoI-6MPK_Up and SmaI-6MPK_Do were used to amplify *TK0664* from the *T. kodakarensis* KOD1 genome. Then, *TK0664* and the vector pUFH were digested by SmaI (S) and XhoI (X) and ligated after a gel purification step, to form plasmid pUPH where *pyrF* is replaced by *TK0664*. The restriction sites BamHI (B) and KpnI (K) were conserved to enable cloning of the homologous regions in pUPH.

Plasmid pUFH served as a starting point to construct our new suicide vector [31]. This plasmid bears two selection markers: the *HMG-CoA* gene of *Pyrococcus furiosus*, encoding 3-hydroxy-3-methylglutaryl coenzyme A reductase, to reduce the inhibitory effects of simvastatin [41]; and the *pyrF* gene of *T. barophilus*, encoding the Orotidine-5’-monophosphate (OMP) decarboxylase, to confer sensitivity to 5-FOA [28,42]*.* In this study, *pyrF* was replaced by *TK0664* amplified from *T. kodakarensis* KOD1 (primers XhoI-6MPK_Up and SmaI-6MPK_Do). To achieve this, the XhoI and SmaI sites were used and the resulting plasmid was named pUPH (Figure 1).

**Figure 2:**
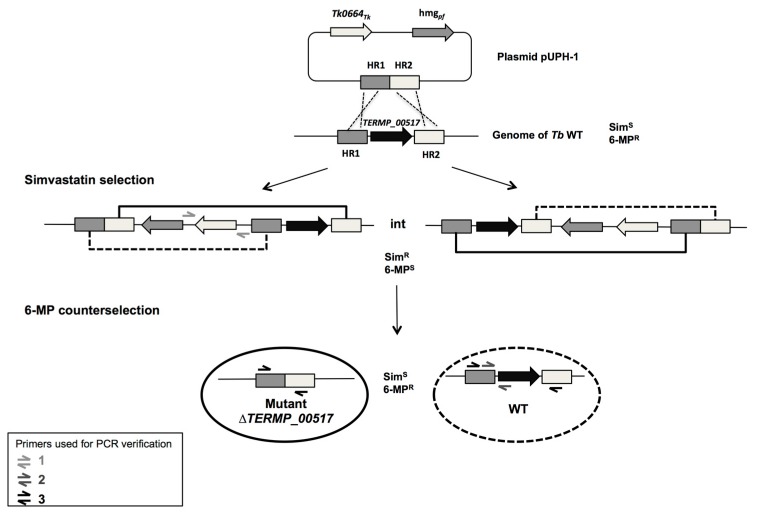
Schematic deletion diagram of *TERMP_00517*. The plasmid pUPH-1 was constructed by ligation of homologous regions (HR) flanking *TERMP_*00517 inside pUPH and used to transform *T. barophilus* MP. After a first homologous recombination (pop-in), cells containing the integrated plasmid were selected with simvastatin (int). The pair of primers 1 was used to verify plasmid integration at this step. Then, intermediated cells were spread on 6-MP to get the second recombination event (pop-out), resulting in plasmid excision. This could lead either to gene deletion or to a WT genotype, depending on the recombination site (full line or dashed line respectively). Primer pairs 2 and 3 are used to validate successful creation of a mutant strain.

According to Thiel et al. (2014), the flanking regions of the targeted gene were amplified sequentially with an overlap extension to form a fragment of 2 kb [31]. Then, this fragment was inserted between the BamHI and KpnI sites of pUPH. The primers 6MPb_1Up, 6MPb_1Do, 6MPb_2Up and 6MPb_2Do (Table 2) were used to delete *TERMP_00517* encoding xanthine-guanine phosphoribosyltransferase. All primers used in the present study are detailed in Table 2. The deletion pathway and pop-in/pop-out recombination steps are described in Figure 2.

### 2.4. Transformation of Thermococcus barophilus

The transformation protocol used in this study was identical to that described by Thiel et al. (2014) [31]. No CaCl_2_ cell treatment was required for the transformation and sulfur was omitted from the TRM medium during the 6 h of cell pre-incubation at 85 °C. Then, the cells were harvested by centrifugation (8000× *g*, 6 min) concentrated in 200 µL of fresh TRM without sulfur; and kept on ice for 30 min. An aliquot of 4–5 µg of plasmid DNA was added to the cells, and the mixture was incubated for 1 h on ice. The heatshock step was carried out at 85 °C for 10 min and was followed by an incubation of 10 min on ice. Finally, the transformants were used to inoculate 20 mL of fresh TRM medium supplemented with sulfur and incubated at 85 °C for 18 h.

After transformation, the cells that had integrated the plasmid into their chromosome were selected on solid medium (Pop-in recombination, Figure 2). At 85 °C, phytagel maintains TRM plates in a solid state (Sigma, 10 g/L). Cells were harvested by centrifugation (8000× *g*, 6 min), resuspended in 100 µL of fresh TRM before spreading on plates containing simvastatin (final concentration 2.5 µg/mL). The plates were then incubated for five days at 85 °C.

A second step was needed to excise the targeted gene (pop-out recombination, Figure 2). This counterselection was performed on plates containing TRM, supplemented with 6-MP (final concentration 100 µM). The strains growing on these plates were resistant to 6-MP and sensitive to simvastatin, since the plasmids had been excised. A PCR was performed, and the PCR products sequenced to examine the different mutants.

### 2.5. DNA Extraction and Purification

First, overnight cultures were centrifuged at 8000× *g* for 8 min. Then, the pellet was suspended in 300 µL of TE (100 mM of Tris-HCl pH8, 50 mM of NaCl, 50 mM of EDTA pH 8). To ensure cell lysis, 40 µL of SDS (10%), 40 µL of Sarkosyl (10%) and 20 µL of proteinase K (20 mg/mL) were added. The cell suspension was incubated for 1 h at 55 °C. Then, 20 µL of RNase A (50 mg/mL) and 200 µL of lysis buffer (GeneJet Genomic DNA purification kit, Thermofisher Illkirch, France) were added. The mix was put into a purification column and centrifuged at 6000× *g* for 1 min. Two successive cleaning steps were carried out, with 500 µL of cleaning solution and 1 min of centrifugation at 12,000× *g*. To remove any trace of ethanol, the suspension was centrifuged (12,000× *g*, 3 min). Then, the column was placed in a clean tube and 200 µL of sterile water used for elution after centrifugation at 12,000× *g* for 1 min.

## 3. Results

### 3.1. Sensitivity of T. barophilus MP to the Purine Analog 6-MP

**Figure 3:**
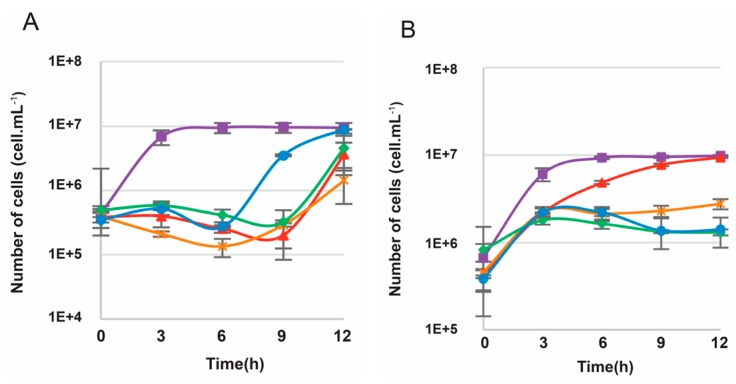
Growth curves of: *T. kodakarensis* KOD1 (**A**); and *T. barophilus* MP (**B**). The strains were cultivated at 85 °C and 0.1 MPa in TRM medium containing different concentrations of 6-MP: 0 µM (■), 10 µM (**▲**), 50 µM (**×**), 100 µM (♦) and 250 µM (•).

To verify if wild type (WT) *T. barophilus* cells were sensitive to 6-MP, growth experiments were conducted on liquid TRM media containing 6-MP at concentrations ranging from 0 to 250 µM (Figure 3A). Growth of the WT strain was slightly impacted in the presence of 10 µM 6-MP, with a slight effect on the growth rate but not on the growth yield. As shown in Figure 3A, at a minimum concentration of 50 µM 6-MP, an inhibitory effect on growth was observed, with a halt in growth between 3 and 12 h (Figure 3A) that could even last until 24 h (data not shown). The same inhibitory effect was observed with higher concentrations of 6-MP (100 and 250 µM).

To make a comparison with *T. kodakarensis*, in which 6-MP is used as a selection marker in genetic manipulations, we performed the same growth experiments in *T. kodakarensis* KOD1, with the same range of concentrations of 6-MP (Figure 3B). Regardless of the concentration used, growth inhibition was observed, but with a resumption of growth beyond 6 or 9 h of incubation, which might possibly be due to the growth of resistant cells.

As genetic manipulations are mainly carried out on solid culture media, we tested whether *T. barophilus* is sensitive or not to 6-MP compared with *T. kodakarensis*. Therefore, we did spot tests to assess the growth of *T. barophilus* on TRM solid medium supplemented with 6-MP concentrations ranging from 0 to 250 µM, followed by the incubation of the spotted plates for five days at 85 °C under anaerobic conditions. When comparing *T. barophilus* with *T. kodakarensis*, the results highlighted a clear difference at 100 µM, with no growth in *T. kodakarensis* (data not shown), whereas *T. barophilus* cells were able to grow, even at 250 µM, but not at 500 µM. According to the results obtained, the *T. barophilus* WT strain does not appear to be very sensitive to 6-MP.

It is known that the sensitivity of *T. kodakarensis* to 6-MP is linked to the *TK0664* gene encoding a hypoxanthine/guanine phosphoribosyltransferase [37]. By analyzing the genome of *T. barophilus* [43], we identified the *TERMP_00517* gene encoding a hypoxanthine/guanine phosphoribosyltransferase. The TK0664 gene product is composed of 214 amino acids), while the *TERMP_00517* gene product is composed of 215 aa, these two proteins share 80.5% identity and 90.2% similarity in the peptide sequence. Given the degree of similarity between the two proteins, it was questioned whether the product of the *TERMP_00517* gene is responsible to some extent for the relative sensitivity of *T. barophilus* to 6-MP. To test this, *TERMP_00517* deletion experiments were performed.

### 3.2. Construction of the ∆TERMP_00517

**Figure 4:**
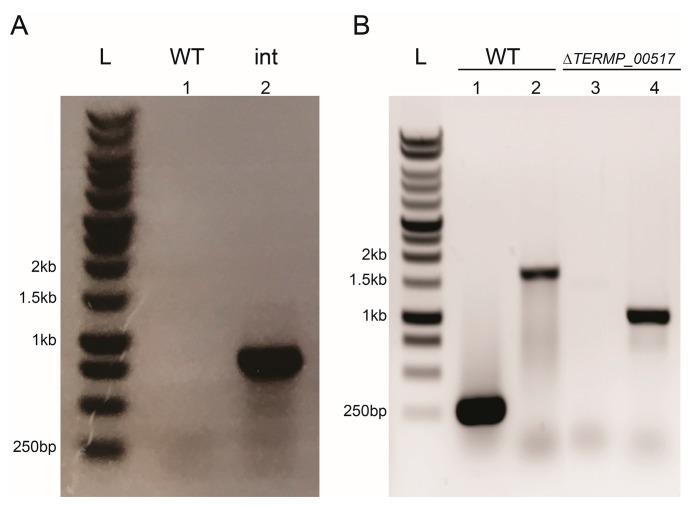
PCR gel migration of mutant strain constructions. (**A**) *TK0664* gene amplification realized with primers XhoI-6MPK_Up and SmaI-6MPK_Do (Primer pair 1, Figure 2) to verify the genotype of pUPH after integration into the *T. barophilus* genome (int, well 2, 819 bp). (**B**) *TERM_00517* gene amplification realized with primers Verif-∆6MPB_Up and Verif-∆6MPB_Do (wells 1 and 3, Primer pair 2, Figure 2) and primers 6MPB_verif_YM_Up and 6MPB_verif_YM_Do (wells 2 and 4, Primer pair 3, Figure 2).

After several unsuccessful attempts to delete *TERMP_00517* with the pUFH suicide vector [31], the new construct pUPH (Figure 1) containing the *TK0664* marker cassette was used to clone the flanking area of this targeted gene. The resulting plasmid (pUPH-1) served in the transformation of *T. barophilus* MP using simvastatin as a marker for the pop-in recombination step (Figure 2). Several transformants were checked for plasmid integration by PCR after genomic DNA extraction. As shown in Figure 4A, the gene *TK0664* was present in the transformants and absent from WT *T. barophilus*. Then, selected clones were spread on solid TRM medium supplemented with 100 µM 6-MP. After five days of incubation at 85 °C, among the hundreds of clones obtained, more than sixty were streaked on solid TRM medium with or without added simvastatin (2.5 µg/mL). This was done to validate the pop-out recombination step or the plasmid excision from the genome and to compare the efficiency of 6-MP with 5-FOA [31]. Thus, more than forty clones were sensitive to simvastatin, among which twenty-four were checked for targeted gene deletion. Finally, PCR controls made it possible to identify six clones as ∆*TERMP_00517* (Figure 4B). One of these strain mutants was selected to be used as a host strain in further genetic experiments.

### 3.3. TERMP_00517 Is Responsible for 6-MP Sensitivity in Thermococcus barophilus

**Figure 5:**
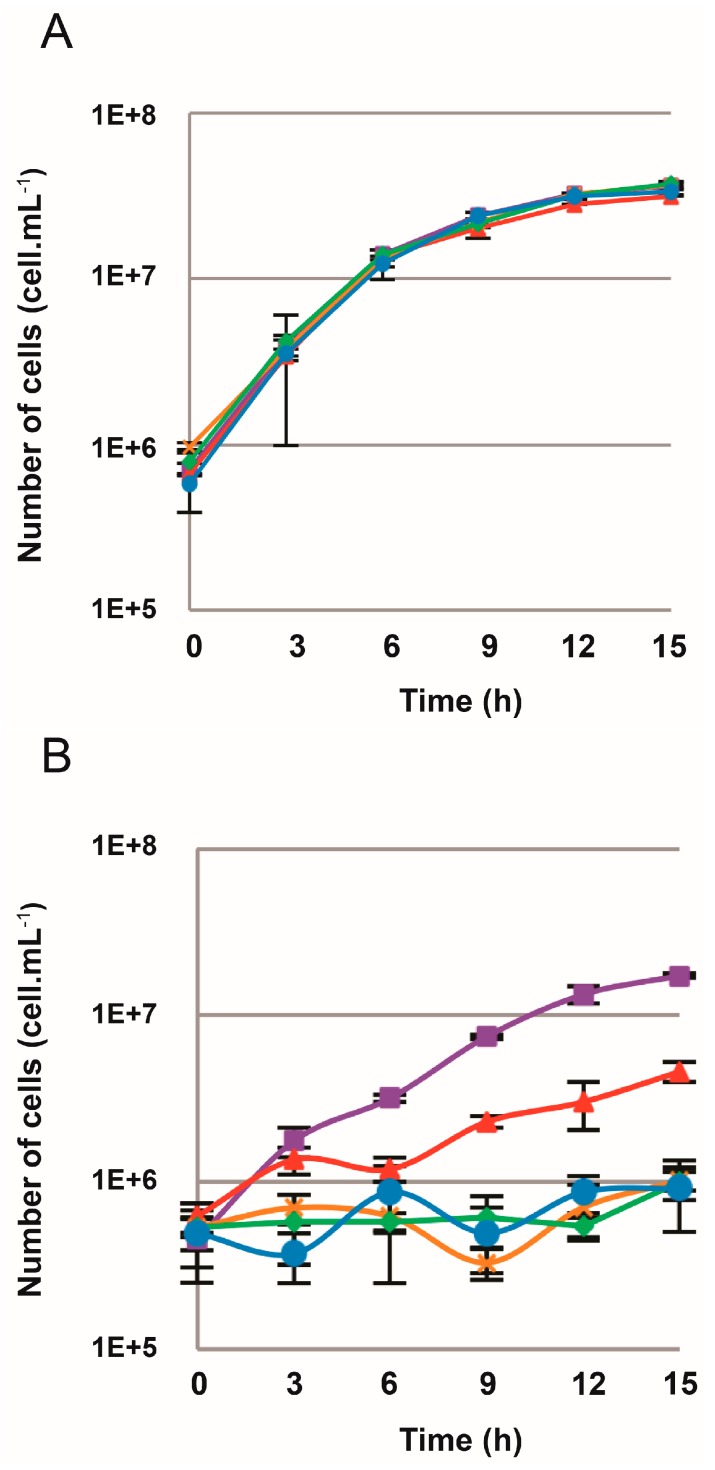
Growth curves of: *T. barophilus ∆TERMP_00517* (**A**); and *T. barophilus* ∆*TERMP_00517*::pUPH-1 (**B**). The strains were cultivated at 85 °C and 0.1 MPa in TRM medium containing different concentrations of 6-MP: 0 µM (■), 10 µM (**▲**), 50 µM (**×**), 100 µM (♦) and 250 µM (•).

Growth monitoring was carried out with the Δ*TREMP-00517 T. barophilus* mutant on liquid TRM medium with concentrations of 6-MP ranging from 0 to 250 µM. Results showed that the mutant was resistant to 6-MP regardless of the concentration used, and demonstrated that the *TREMP_00517* gene was responsible for the relative sensitivity to 6-MP in *T. barophilus* (compared with *T. kodakarensis*) and that its deletion did not affect the viability of the strain under selected culture conditions (TRM medium, 85 °C, anaerobic conditions) (Figure 5A).

The mutant Δ*TREMP_00517 T. barophilus* was transformed with the plasmid pUPH-1 bearing the flanking genes of the *TERMP_00517* and *TK0664* genes. The integration of this plasmid was made upstream or downstream of the TERMP_00517 deleted region, the epictopic complemented mutant resistant to simvastatin was grown in liquid TRM medium supplemented with 6-MP up to 250 µM (Figure 5B). This strain was slightly sensitive to 10 µM 6-MP and its growth was completely inhibited at 50 µM 6-MP, showing that the *Tk0664* gene confers sensitivity to 6-MP (Figure 5B) much more effectively than the *TERMP_00517* gene, as shown in WT *T. barophilus* (Figure 3A).

Thus, deletion of *TREMP_00517*, the gene encoding xanthine-guanine phosphoribosyltransferase, confers resistance to 6-MP along with a higher sensitivity through complementation with the *TK0664* gene.

### 3.4. Efficiency of the New Counterselection Marker

To assess the efficiency of this new counterselection system, pop-in and pop-out recombinations were made for different target genes. Due to the randomization of the pop-out step that can either lead to the WT strain or to gene deletion, several clones were screened by PCR to verify the veracity of the mutation. However, it was also necessary to check for the absence of the plasmid from the strain genome. For *T. barophilus*, 5FOA has been previously used as a drug for the counterselection step [31] but a high rate of false positive clones (still containing the plasmid after growth on 5FOA plates) was recurrently observed. Consequently, colonies were re-streaked on TRM-simvastatin plates to sort out the ones that had really lost the plasmid. This false-positive screening is time-consuming because it requires another 3–5 days of incubation at 85 °C before screening for potential mutants is possible. The use of 6-MP could therefore strongly reduce the processing time of mutant constructions, in addition to expanding the utility of *T. barophilus* as a genetic tool.

Overall, with 5-FOA, 726 clones were screened and 625 were false positives (86%). With 6-MP, as described above, the first construction (∆*TERMP_00517*) gave a rate of 37% false positives, which represents a strong decrease. For the next genetic deletions realized with the strain ∆*TERMP_00517*, among the 326 clones screened, none were false positives (0%). This difference is highly significant (*t*-test, *p*-value < 10^−22^) and clearly demonstrates the improvement of the pop-out recombination step or the counterselection step with 6-MP.

### 3.5. Deletion of polB or rnhB Is Not Essential for Viability in Thermococcus barophilus

The construction of effective genetic tools in *T. barophilus*, a deep sea hydrothermal vent Thermococcales, completes the range of approaches used in our laboratory to study the cellular processes of *Thermococcales* essential for adaptation and genomic maintenance under extreme temperature and/or high hydrostatic pressure conditions of deep sea hydrothermal vents.

Among the different cellular processes being studied with this new genetic tool, a special emphasis is being put on genome replication, centering on the functional characterization of the Thermococcales (e.g., *Pyrococcus abyssi* and *T. barophilus*) replication complex. Here, the PolB and PolD DNA polymerases found in *Thermococcales* carry out DNA synthesis. It has been shown in a *T. kodakarensis* strain deficient in PolB that this does not affect growth rate [36] and PolB does provide resistance to UV irradiation. In contrast, all attempts to generate mutants deleted in *polD* have been unsuccessful.

**Figure 6:**
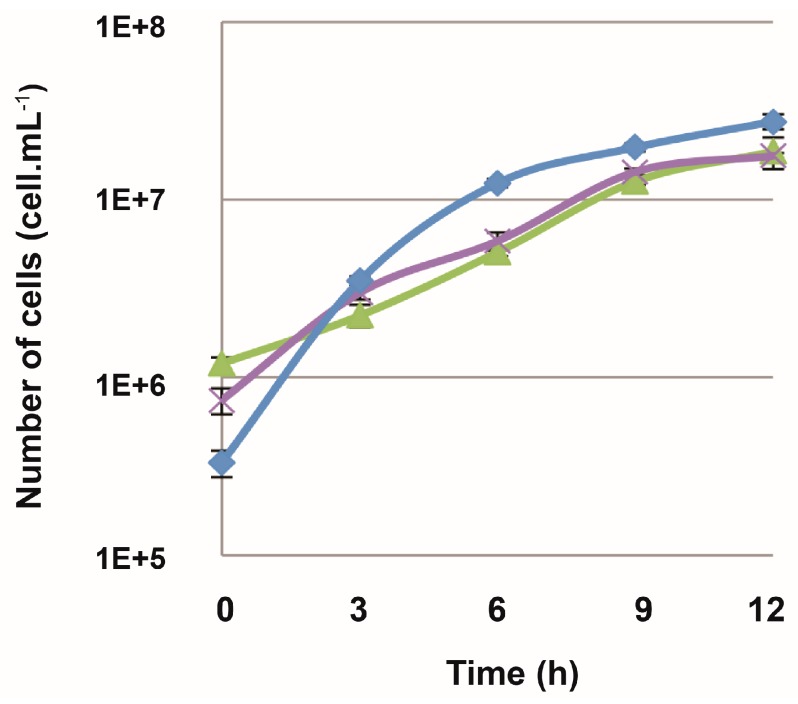
Growth curves of *T. barophilus* ∆*TERMP_00517*, *T. barophilus* ∆TERMP_00517, ∆*TERMP_01623* (*polB*) and *T. barophilus* ∆*TERMP_00517*, ∆*TERMP_00671* (*rnhB*). The strains were cultivated at 85 °C and 0.1 MPa in TRM medium containing different concentration of 6-MP: *T. barophilus* ∆*TERMP_00517*, ∆*rnhB* (**×**), *T. barophilus* ∆*TERMP_00517*, ∆*polB* (**▲**) and *T. barophilus* ∆*TERMP_00517* (parental strain) (♦).

The *polD* and *polB* genes were targeted for deletion in *T. barophilus*, with primers designed (Table 2) to delete the genes *TERMP_01623* (PolB) and *TERMP_01872-TERMP_01873* (large and small subunits of PolD). Plasmids containing upstream and downstream homologous regions of these two deletion targets were inserted in the pUPH plasmid (at KpnI/BamHI restrictions sites). For *polB*, the counterselection on 6-MP made it possible to isolate several clones resistant to 6-MP that had been effectively deleted in the *TERMP_01623* (*PolB*) gene. Conversely, several attempts in deleting *polD* have failed, demonstrating that as for *T. kodakarensis* [39] that PolB is not essential for cell growth (Figure 6) while PolD is and that PolD is sufficient for replicating DNA under the culture conditions used in this study.

DNA replication in all living organisms takes place concurrently on two separate strands. The lagging strand consists of multiple discontinuous segments called Okazaki fragments, whereas the leading strand consists of one large continuous segment. The production of each individual lagging strand by DNA polymerase is primed by a short stretch of RNA. RNases H are enzymes that degrade the RNA portion of RNA/DNA or RNA-DNA/DNA duplexes and RNase HII is likely the key enzyme involved in RNA elimination in Thermococcales [44]. In the same way as for *polB*, primers were designed to delete the *rnhB* gene. Genomic regions upstream and downstream of the targeted gene were PCR-amplified and cloned in the pUPH plasmid. The Δ*TERMP_00517 T. barophilus* strain was transformed by this new construction and the deletion of the mutant in the *rnhB* gene has since been successfully obtained. Thus, this gene does not appear to be essential for the growth cycle as its growth is similar to that of the parental strain (Figure 6). This confirms what has been recently demonstrated in *T. kodakarensis*, where RNase HII individual deletion is not essential for cell growth [38].

## 4. Discussion

In this study, we have shown that the WT *T. barophilus* MP strain is relatively resistant to 6-MP, and the deletion of the *TERMP_00517* gene homologous to the *TK0664* gene makes the strain completely resistant to 6-MP. The epictopic expression of *TK0664* in the *T. barophilus* strain deleted for the *TERMP_00517* gene makes it completely sensitive to 6-MP.

Based on the lethal incorporation of 6-methylpurine in the purine salvage pathway through TK0664 expression, the TK0664/6-MP system has allowed the deletion of multiple genes in different archaea [23,45]. In this study, we demonstrated the relevance of using this counterselection marker in *T. barophilus*. Despite the presence in its genome of the *TERMP_00517* gene encoding xanthine-guanine phosphoribosyltransferase, homologous to *TK0664*, *T. barophilus* has previously been described as insensitive to 6-MP [31]. Here, the results of our study highlighted the influence of this gene on the sensitivity of the WT strain. Indeed, a dose-effect is observed with the WT *T. barophilus* strain but this had totally disappeared from the strain with an impaired *TERMP_00517* gene (Figure 3A and Figure 5A). Moreover, the sensitivity of *T. barophilus* to 6-MP conferred by *TK0664* is much higher than that allowed by *TERMP_00517* (Figure 5B). Although the two gene products share more than 80% identity and 90% similarity, it seems that the xanthine-guanine phosphoribosyltransferase enzyme encoded by *TK0664* gene is more efficient than that encoded by *TREMP_00517* gene in producing sufficient toxic product after 6-MP enzymatic conversion.

It should be mentioned that the system with the *pyrF*/5FOA marker was very useful for initiating genetic manipulations in *T. barophilus* [31], but because the percentage of false positives is too high, the time needed to obtain a mutant is much longer, requiring more liquid culture steps, restreaking on solid media, and PCR checking, which together lengthen the procedure considerably.

With the ∆*TERMP_00517 T. barophilus* strain, the new system gives few or no false positives (less than 15%) after screening on solid TRM supplemented with simvastatin, which substantially reduces the time required to obtain mutants in *T. barophilus*. However, it should be noted that this substantial improvement does not make it possible to skip the PCR verification step to truly verify plasmid excision.

The new pUPH suicide vector bearing simvastatin (*hmg-CoA*) resistance and 6-MP (*TK0664*) sensitivity markers that was constructed and used in this study for *T. barophilus* has already allowed individual deletion of the genes *TERMP_00517*, *polB* and *rnhB* in *T. barophilus.* Here, the results obtained on genes encoding DNA polymerases corroborate the results obtained in *T. kodakarensis* [39]: PolD is likely the essential replicative enzyme and PolB could play a role in DNA repair and/or recombination and an alternative role in DNA replication [39]. As demonstrated in this study, loss of *rnhB* from *T. barophilus* had no effect on growth rate. This corroborates a recent study showing that RNase HII can be deleted in *T. kodakarensis* with no discernible effects on viability and growth [38]. However, it seems that RNase HII must collaborate with Fen1 (a flap endonuclease) to maintain viability in the absence of GAN (for GINS-associated nuclease) in *T. kodakarensis* [38].

## 5. Conclusions

This improvement enlarges the number of genetic selection markers for *T. barophilus*, which is particularly valuable at present to pursue investigations on piezophilic adaptation in this deep-sea hydrothermal vent archaeon [20]. As illustrated in this study, *T. barophilus* can also serve as a model for the genetic and physiological studies of other cellular processes such as the genomic maintenance under high temperature and high pressure, corresponding to the environmental conditions found in deep sea hydrothermal vents.

## Figures and Tables

**Table 1:** *Thermococcus barophilus* strains used and constructed in this study.

Strain	Genotype	Parent Strain	References
UBOCC-M-3203	Wild Type	*T. kodakarensis* KOD1	[22]
UBOCC-M-3107	Wild Type	*T. barophilus* MP	[17]
UBOCC-M-3300	TB∆*TERMP_00517*	*T. barophilus MP*	This study
UBOCC-M-3301	TB∆*TERMP_00517*::*TK0664*	*T. barophilus MP*	This study
UBOCC-M-3302	TB∆*TERMP_00517*, ∆*TERMP_01623*	*T. barophilus MP*	This study
UBOCC-M-3303	TB∆*TERMP_00517*, ∆*TERMP_00671*	*T. barophilus MP*	This study

**Table 2:** List of primers used in this study.

Primers Used for Amplification of Flanking Regions of Targeted Gene	Sequence (5′-3′)	Tm (°C)
PolB_1Up	AAAAAAGGTACCGCTTAACATTCCTGACTCCCAGAATCTT	59.4
PolB_1Do	TCTATTTCATTAAATCACCTAATTTCACCCTTTTAAAAATACATGCCCAT	57.9
PolB_2Up	ATTTTTAAAAGGGTGAAATTAGGTGATTTAATGAAATAGAATGAGCAGGA	57.9
PolB_2Do	AAAAAAGGATCCCGGCTTCTGGGGAAACCTCG	60.6
6MPb_1Up	AAAAAACGTACCAAGAAAACCGGAGTTTTAGTGAATACACC	58.4
6MPb_1Do	TCTCATGGAAACATTTAAATGGTTGTGGTATCTTGGACAAGAAGAAAA	59.5
6MPb_2Up	TTGTCCAAGATACCACAACCATTTAAATGTTTCCATGAGAAAAATGAAATGCAAAAA	60.3
6MPb_2Do	AAAAAAAGATCTCTCGCTCTAAAGGAGCTTTCAACA	56.0
RHII_1Up	AAAAAAGGTACCCGGTACCCTGATAAAGAAGGCATC	59.4
RHII_1Do	TTAGTATTCAGGAAATGAGGACTCTTGAGGTTCTTTCTCTTCGGT	61.3
RHII_2_Up	AGAGAAAGAACCTCAAGAGTCCTCATTTCCTGAATACTAACGTTCCG	63.0
RHII_2_Do	AAAAAAAGATCTACGACTCATGATGTTAATCCTCTTACAGAG	57.4
**Primers Used to Check Mutants**	**Sequence (5′-3′)**	**Tm (°C)**
PolB_Up	GTCAGCTATGAGCTCGTGAAGAGTTAT	59
PolB_Do	AGAAGAGCGTAAATGTAAGGCTGGA	59
6MPk_Up	AAAAAACTCGAGCCCGTCCAAGCTACCACTCCC	69
6MPk_Do	AAAAAACCCGGCTTCAAAACCCAGCCAAACAACACCC	71
6MPb_Up	ACCTCAGACATCTCTGCTGCTTG	60
6MPb_Do	GCCCCGATAAGTGCTGAAAGATACA	60
RHII_Up	GAGGAATCGCTGAAGTTTTACTGGG	59
RHII_Do	CTACAAAGAGCATGGCGAATTTCCG	60

Tm: Melting temperature.
